# The Medical Basis for the Photoluminescence of Indocyanine Green

**DOI:** 10.3390/molecules30040888

**Published:** 2025-02-14

**Authors:** Wiktoria Mytych, Dorota Bartusik-Aebisher, David Aebisher

**Affiliations:** 1 English Division Science Club, Medical College, The Rzeszów University, 35-310 Rzeszów, Poland; wiktoriamytych@gmail.com; 2 Department of Biochemistry and General Chemistry, Medical College, The Rzeszów University, 35-310 Rzeszów, Poland; dbartusikaebisher@ur.edu.pl; 3 Department of Photomedicine and Physical Chemistry, Medical College, The Rzeszów University, 35-310 Rzeszów, Poland

**Keywords:** photoluminescence, indocyanine green, fluorescence, near-infrared, surgery

## Abstract

Indocyanine green (ICG), a near-infrared (NIR) fluorescent dye with unique photoluminescent properties, is a helpful tool in many medical applications. ICG produces fluorescence when excited by NIR light, enabling accurate tissue visualization and real-time imaging. This study investigates the fundamental processes behind ICG’s photoluminescence as well as its present and possible applications in treatments and medical diagnostics. Fluorescence-guided surgery (FGS) has been transformed by ICG’s capacity to visualize tumors, highlight blood flow, and facilitate lymphatic mapping, all of which have improved surgical accuracy and patient outcomes. Furthermore, the fluorescence of the dye is being studied for new therapeutic approaches, like photothermal therapy, in which NIR light can activate ICG to target and destroy cancer cells. We go over the benefits and drawbacks of ICG’s photoluminescent qualities in therapeutic contexts, as well as current studies that focus on improving its effectiveness, security, and adaptability. More precise disease detection, real-time monitoring, and tailored therapy options across a variety of medical specialties are made possible by the ongoing advancement of ICG-based imaging methods and therapies. In the main part of our work, we strive to take into account the latest reports; therefore, we used clinical articles going back to 2020. However, for the sake of the theoretical part, the oldest article used by us is from 1995.

## 1. Introduction

### 1.1. Biochemical Properties of Indocyanine Green

**Figure 1:**
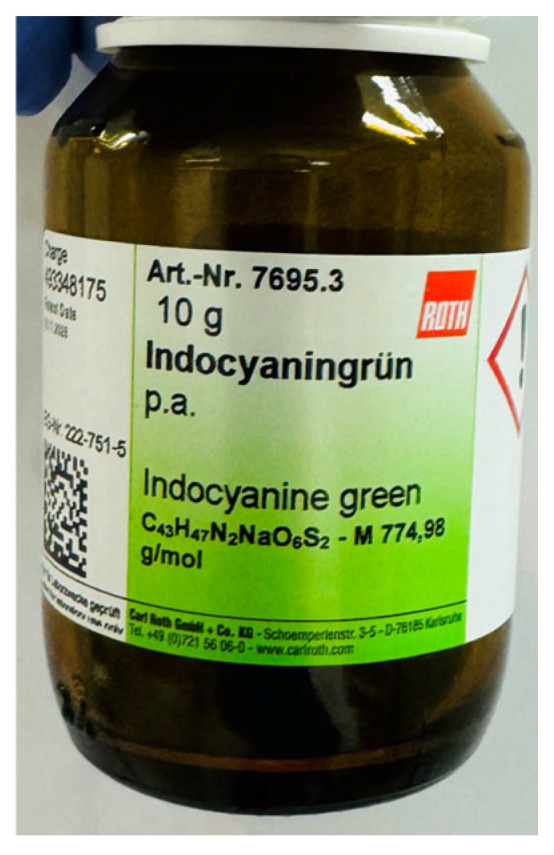
Indocyanine green (manufactured by Carl Roth (Karsruhe, Germany). Original photography conducted by co-authors.

The scientific and medical communities have shown a great deal of interest in indocyanine green (ICG) (Figure 1), a water-soluble synthetic dye, due to its unique chemical properties and numerous applications [1]. Initially created in the 1950s, it is primarily used for diagnostic purposes, ranging from liver and heart function tests to ocular imaging [2]. As a fluorophore with biological activity, this compound serves both diagnostic and therapeutic functions. This section explores the chemistry, biological activity, and applications of the dye, focusing on medical imaging and clinical diagnostics [3]. The mentioned compound belongs to the heptamethine cyanine dye subclass, characterized by conjugated double-bond systems that enhance its optical properties [4]. The molecular structure consists of a core aromatic ring system and two indole or indoline rings joined by a polymethine chain, contributing to its vibrant color and ability to absorb and release light at specific wavelengths [5].

When activated, it absorbs near-infrared (NIR) light at approximately 780 nm and fluoresces in the 800–850 nm range. The NIR absorption and fluorescence are particularly advantageous for medical imaging, as they allow for deeper tissue penetration with less dispersion than visible light [6]. The addition of a sulfonate group (-SO₃Na) enhances the molecule’s water solubility, making it suitable for intravenous delivery. After injection, the compound is rapidly absorbed into the bloodstream and processed by the liver. The pharmacokinetics of the dye are influenced by various factors such as liver function, blood circulation, and the delivery method [7]. During medical procedures like liver function tests and coronary angiography, the dye’s clearance rate provides valuable insights into organ function, with the liver serving as the primary route for elimination [8]. After intravenous administration, it travels to the liver, where it is excreted as bile.

**Figure 2:**
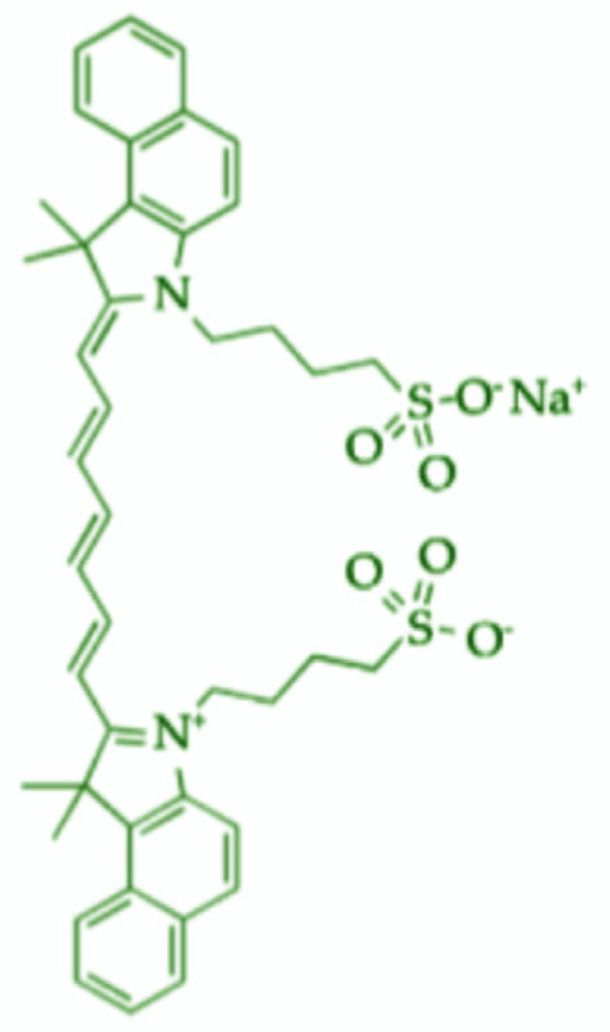
Indocyanine green chemical structure. Original work created by co-authors in bioRender (Toronto, ON, Canada).

The excretion through the biliary system is crucial for evaluating liver function. ICG (Figure 2) typically has a short half-life before leaving the bloodstream, enabling real-time physiological studies, which is beneficial for imaging applications [9]. However, individuals with liver disease experience a longer half-life and delayed clearance due to impaired liver function [10,11]. This clearance is often used as an indirect indicator of liver health, particularly in liver transplantation scenarios. While the liver is the main elimination route, a very small amount of the compound is removed by the kidneys. ICG’s low toxicity profile, coupled with its rapid elimination from the body, minimizes the risk of accumulation and toxicity [12,13]. Though adverse effects are rare, they can include fever, nausea, or allergic reactions, with severe reactions like anaphylaxis being exceptionally uncommon. In cases of severe liver failure or biliary obstruction, administration must be conducted with care, and caution is advised for individuals allergic to iodine or iodine-based contrast agents [14,15]. ICG is typically stored in a powdered form until reconstituted in a sterile solution. Prolonged exposure to light can degrade its optical properties, so it should be kept in dark or opaque containers. The solution is prepared fresh in clinical settings to maintain its fluorescence and absorption stability [16,17]. Additionally, the pH and concentration of the solution have a slight impact on its stability. Fluorescence imaging remains the most prominent use of ICG. Due to its near-infrared emission, it offers high-contrast, real-time visualization of tissues, blood vessels, and organs [18].

### 1.2. Synthesis of Indocyanine Green

ICG is a cyanine dye widely used in photothermal treatments and medical diagnostics. Its synthesis begins with the condensation of a carbonyl compound (usually an aldehyde) and a heterocyclic amine (such as a quinoline or pyrrole derivative), which forms a polymethine bridge through alternating single and double bonds [19]. The presence of sulfonate groups enhances the dye’s water solubility, and these are introduced via sulfonation, often under controlled conditions using reagents like sulfur trioxide or chlorosulfonic acid. After sulfonation, the product is purified, and further functional groups, including iodine atoms, are incorporated to adjust its absorption properties [20,21]. ICG analogs can be created by modifying various components of the dye structure, including the length of the polymethine chain and the heterocyclic rings, to alter fluorescence and absorption characteristics. The spectral properties are fine-tuned to optimize its application in bioimaging, as the near-infrared range (780–800 nm absorption and 830 nm emission) facilitates deeper tissue penetration than visible light [22,23]. The aggregation of ICG in solution, known as aggregachromism, can significantly shift its absorption and emission spectra, particularly when the concentration is high or in the presence of aggregation-promoting factors [24]. However, the dye can maintain its structure and fluorescence characteristics when dissolved in organic solvents, avoiding the spectral shifts seen in aqueous solutions. Aggregation is less likely in non-polar solvents, which generally enhance fluorescence performance [25]. ICG’s susceptibility to photobleaching, wherein prolonged light exposure causes irreversible fluorescence loss, is another important consideration. Factors like solvent polarity, oxygen presence, and ICG concentration influence the rate of photobleaching [26]. These photophysical properties are critical for ICG’s role in medical imaging, including fluorescence-guided surgery and angiography.

### 1.3. Photoluminescence of Indocyanine Green

**Figure 3:**
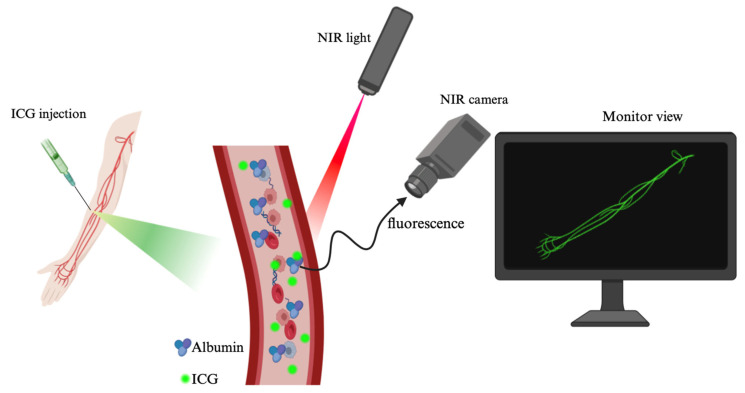
Photoluminescence mechanism. Original work created by the co-authors in bioRender (Toronto, ON, Canada). The colors of the arrows are random and have no scientific significance.

Photoluminescence refers to the emission of light after a substance absorbs photons. Upon absorbing light, the compound’s electrons are excited to a higher energy state. As they return to their ground state, they release light, resulting in fluorescence or phosphorescence [27,28]. The dye’s photoluminescent properties are central to its use in medical imaging. The fluorescence process begins when ICG absorbs photons in the NIR spectrum (around 780 nm) [29,30]. The energy from the absorbed photons excites electrons in the polymethine chain, elevating them to a higher energy state. Internal conversion follows, where the excited electron loses some energy through vibrational relaxation but remains in an excited state. Fluorescence emission occurs when the electron returns to the ground state, releasing energy as photons. The emitted light has a longer wavelength than the absorbed light, and ICG typically has a fluorescence lifetime in the millisecond range [31,32,33,34,35,36,37]. The fluorescence intensity of ICG is affected by its binding to plasma proteins, particularly albumin, altering its emission spectrum and intensity (Figure 3) [38,39,40]. Furthermore, the dye’s photoluminescence makes it highly valuable for vascular imaging, where real-time tissue observation is required. Due to its absorption and emission in the NIR spectrum, ICG penetrates biological tissues with minimal scattering compared to visible light [41,42,43].

NIR light can image organs, blood vessels, and tumors more deeply without being overly obstructed by tissue due to its low tissue dispersion. ICG is therefore an ideal technique for real-time imaging of internal tissues during medical procedures such as liver function assessment, heart diagnostics, and fluorescence-guided surgery (FGS) [44,45]. Using NIR fluorescence reduces autofluorescence in tissues, which often occurs with visible light. This enhancement of the contrast between the dye and surrounding tissue allows for more accurate and clear imaging. ICG is frequently utilized in clinical practice for intraoperative imaging during surgeries, including blood flow visualization, malignant tissue identification, and tumor excision guidance [46]. Additionally, ICG’s photoluminescence can help identify lymphatic drainage patterns, which can help with sentinel lymph node mapping, especially in cases of breast and melanoma cancer. Additionally, FGS uses the dye’s photoluminescence to increase tumor removal precision and lower the possibility of leaving cancerous tissue behind [47,48]. Beyond imaging, ICG’s fluorescence-emitting properties are also being investigated for medicinal uses, such as photothermal therapy, in which NIR light can activate it to produce heat and kill cancer cells only [49,50]. As more study is conducted, the photoluminescent qualities of ICG should pave the way for new developments in targeted therapeutics and non-invasive diagnostics, which will enhance the identification and management of several illnesses. In a study from 2020, 23 patients had their primary and metastatic liver cancers surgically removed with the use of a new optical imaging device that combines visible multispectral imaging with NIR-I (700–900 nm) and NIR-II (1000–1700 nm) fluorescence. The findings demonstrated that NIR-II imaging performed better than NIR-I imaging in several areas, including the tumor detection rate (56.41% vs. 46.15%), the tumor-to-normal-liver-tissue signal ratio (5.33 vs. 1.45), and tumor detection sensitivity (100% vs. 90.6%). According to these results, image-guided surgery may be improved by combining NIR-I and NIR-II imaging with the right fluorescent probes, which could lead to better clinical results for liver tumor resections [51].

### 1.4. Indocyanine Green Incorporating Nanoparticles

Nanoparticles that incorporate ICG are a unique approach for medical diagnostic, imaging, and therapeutic applications. By encapsulating ICG in nanoparticles, researchers and medical experts hope to enhance its fluorescence and biological compatibility while enabling the targeted delivery of drugs, imaging agents, or other therapeutic substances [52,53]. These nanoparticles can optimize ICG’s bio-distribution, stability, and efficiency while also opening new avenues for targeted therapy, nanomedicine, and imaging techniques. Within in vivo settings or when exposed to light (photobleaching), ICG is known to degrade rapidly [54]. Encapsulating ICG in nanoparticles can increase its stability, allowing for more reliable and long-lasting imaging and therapeutic effects. Nanoparticles can operate as platforms that improve the fluorescence properties of ICG by protecting it from environmental factors like oxygen that might otherwise cause it to fade [55,56]. They can also aid in sustained release, which eventually makes consistent imaging possible. Functionalizing nanoparticles with certain ligands or antibodies enables the targeting of particular cells or organs (Table 1). ICG can be carefully applied to tumors in targeted cancer imaging and treatment, increasing the accuracy of the diagnosis and the effectiveness of the treatment [57]. When ICG is encapsulated in nanoparticles for regulated or triggered release, there is greater control over the length of time and site-specificity of imaging or treatment. In medication administration systems where exact dosing is necessary, this feature is very important [58,59].

ICG-incorporating nanoparticles combine the diagnostic power of ICG with the therapeutic promise of nanotechnology. After being encased in nanoparticles, ICG maintains its ability to absorb near-infrared light and emit fluorescence. Continuous and prolonged fluorescence imaging is made possible by the nanoparticles’ ability to protect ICG against environmental degradation and photobleaching [72]. These nanoparticles are used in real-time surgical guidance, vascular imaging, and tumor detection, particularly in fluorescence-guided operations and non-invasive imaging techniques. ICG’s ability to visualize tumor vasculature, tissue perfusion, and blood flow is significantly enhanced by nanoparticle carriers. By functionalizing them with ligands, antibodies, or small chemicals that bind to specific receptors on the surface of cancer cells or tissues, ICG-loaded nanoparticles can target sick areas—particularly tumors—specifically [73,74]. ICG can be utilized in photothermal treatment (PTT), a therapeutic technique in which ICG builds up at the tumor site and absorbs near-infrared light, creating heat that kills tumor cells, due to its strong absorption capabilities. When ICG is encapsulated in nanoparticles, it can be more precisely localized to the tumor site, reducing the risk of damaging neighboring healthy tissues [75]. ICG-loaded nanoparticles’ dual therapeutic (drug administration or photothermal therapy) and diagnostic (fluorescent imaging) properties make them ideal for theranostic applications, which integrate imaging and therapy into a single treatment platform. ICG is not frequently used in photodynamic therapy, but its incorporation into nanoparticles has opened new applications for PDT [76,77]. When exposed to light, ICG can release reactive oxygen species (ROS), which can kill cancer cells or infections. By encapsulating ICG in nanoparticles, its distribution may be more accurately controlled and its photosensitizing efficacy enhanced. ICG-incorporating nanoparticles combine the advantages of nanotechnology with the unique optical properties of ICG to improve medical imaging, therapy, and diagnostics [78]. Their use in theranostics, FGS, and targeted drug delivery has great promise for advancing precision medicine, particularly in the treatment of cancer. Despite ongoing concerns about safety, stability, and regulatory approval, more research and the development of these innovative nanomedicines is likely to lead to new discoveries.

### 1.5. BODIPY and FITC vs. ICG

Fluorescent dyes like FITC (Fluorescein Isothiocyanate), BODIPY (Boron-dipyrromethene), and ICG have become essential tools in biological imaging, but each comes with its own set of advantages and challenges. FITC is widely used due to its high fluorescence yield and ease of conjugation with biomolecules, making it a popular choice in cellular imaging [79]. However, FITC has significant drawbacks, particularly its pH sensitivity, which can lead to fluctuations in fluorescence intensity depending on the local environment. This makes it less reliable in applications where the pH might vary, such as live-cell imaging. Moreover, FITC suffers from relatively low photostability, which means that prolonged imaging can lead to photobleaching, diminishing the quality of the results over time [80]. BODIPY dyes, on the other hand, are known for their superior photostability and bright fluorescence, which make them ideal for long-term imaging studies. They also have a wide range of tunable emission wavelengths, allowing for more flexibility in multi-color imaging. However, BODIPY dyes can be prone to aggregation in certain conditions, which can alter their fluorescence properties and lead to inconsistent results [81,82]. Additionally, while BODIPY dyes are photostable, their narrow absorption and emission spectra can limit their versatility in complex imaging setups that require multiple dyes with broad spectral ranges. In contrast, ICG has emerged as a promising alternative, particularly for in vivo imaging [83,84]. ICG’s major advantages lie in its biocompatibility, strong near-infrared fluorescence, and minimal toxicity, which make it particularly useful for clinical and diagnostic applications, such as the real-time monitoring of tissue perfusion and the detection of tumors [85]. The near-infrared emission of ICG allows for deeper tissue penetration and reduced background interference, which is critical for high-resolution imaging in living organisms. Furthermore, ICG exhibits good stability in physiological conditions, allowing for reliable imaging in various biological environments. However, ICG is not without its challenges [86]. It is known to be rapidly cleared from the bloodstream, which can limit the time window for imaging in some applications. Additionally, its fluorescence can suffer from photobleaching under prolonged exposure to light, especially in high-intensity settings [87]. Moreover, while ICG is highly useful in the near-infrared spectrum, its absorption and emission properties may not be ideal for every imaging scenario, particularly when a broader spectrum of dyes is required for multiplexed imaging [88]. Despite these limitations, ongoing advancements in dye chemistry and imaging technologies continue to improve the performance and versatility of ICG and similar near-infrared dyes, making them increasingly valuable tools in both research and clinical settings.

## 2. Indocyanine Green in Medicine

### 2.1. General Use of ICG in Medicine

In contemporary medicine, ICG is a flexible and extensively utilized diagnostic and imaging tool. Assessing liver function is one of the main clinical applications of ICG. ICG is a great indicator for evaluating hepatic perfusion and liver function since it is mostly eliminated by the liver without being digested [89]. Important details regarding liver function can be gleaned from the rate at which the liver eliminates ICG from the bloodstream. Patients with liver conditions such as cirrhosis, hepatitis, and liver failure are tested for this condition. An extended ICG clearance time indicates hepatic dysfunction. Assessing a patient’s eligibility for liver transplantation can be aided by their ICG clearance. Treatment selections are guided by the test’s insightful information about the liver’s capacity to process and eliminate drugs [90]. ICG is used in indocyanine green angiography (ICGA) to see the eye’s choroid and retina. Ophthalmologists can investigate deeper layers of the eye with ICG’s NIR fluorescence, which is often not possible with conventional visible light procedures like fluorescein angiography. By evaluating macular degeneration, ICGA assists medical professionals in determining the state of the retina’s blood vessels [91,92]. ICG aids in the diagnosis and treatment of diabetic retinopathy by highlighting aberrant blood vessels in the condition. ICG is especially useful for identifying aberrant choroid blood vessel growth, which might be a symptom of conditions such as wet age-related macular degeneration (AMD) [93]. In oncology, ICG is being used more and more for tumor detection and FGS. ICG can be used to highlight lymph nodes, tumors, or aberrant tissue growth when administered intravenously [94]. In cancer procedures, ICG is used for lymphatic mapping, especially in cases of breast and melanoma cancer. The sentinel lymph node, the first lymph node to receive drainage from the tumor, receives the dye after it is injected close to the tumor site and passes through the lymphatic system [95]. By using fluorescence to guide the excision of these lymph nodes, surgeons can increase the precision of cancer staging [96]. ICG can assist in defining tumor boundaries during cancer procedures, guaranteeing the total removal of malignant tissues and lowering the chance of recurrence. ICG’s function in FGS has transformed the field of minimally invasive surgery. During resection procedures, ICG is utilized to visualize colon blood flow to make sure that healthy tissue is maintained and that the residual colon receives enough blood flow [97,98]. ICG can be used to assess coronary artery perfusion during heart surgery, helping surgeons identify areas that need revascularization. During procedures like liver resection or transplantation, surgeons evaluate the liver’s vasculature using ICG. The dye guarantees that vital blood arteries are maintained throughout surgery and aids in seeing the hepatic blood flow [99,100]. ICG is frequently used to evaluate vascular patency and visualize blood vessels. ICG fluoresces make it possible to see blood flow, tissue perfusion, and the vascular network in real-time. In patients with cardiovascular disorders, ICG can be used to measure cardiac output and analyze hemodynamics [101,102]. ICG is used in vascular surgery to help surgeons see blood vessels so they can prevent injury and ensure that tissues receive enough blood. It is particularly useful for endoscopic and bypass surgery [103]. ICG is being utilized more and more in neurosurgery to measure cerebral blood flow. This is crucial for operations like brain tumor resections and brain aneurysm surgeries when there is a risk to brain tissue because of inadequate blood flow [104,105]. The real-time visualization of cerebral blood flow might greatly enhance surgical results by assisting surgeons in avoiding ischemia zones and guaranteeing sufficient perfusion to vital brain locations [106]. After surgery, ICG can be used to evaluate tissue perfusion and make sure that recovery is proceeding as planned. For instance, ICG can be administered during organ transplantation to confirm that the recipient organ is receiving enough blood flow, which can assist in the detection of rejection or complications such as graft failure early on [29]. ICG is used in medical research, specifically in tissue engineering and medication delivery systems, in addition to its clinical applications. ICG is used by researchers to monitor drug distribution in the body, investigate blood flow dynamics, and investigate novel treatments for diseases like cancer, heart disease, and neurological problems. 

### 2.2. ICG Fluorescence in Sentinel Lymph Node Biopsy and Other Breast Cancer Procedures

**Figure 4:**
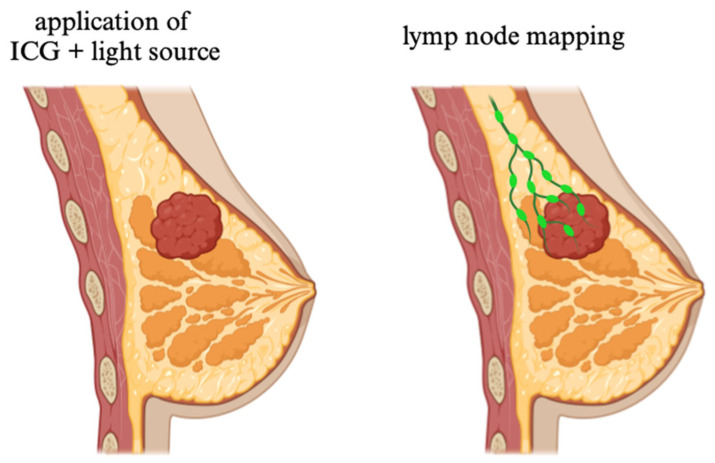
Sentinel node biopsy. Dye injected from the side of the tumor spreads to the sentinel nodes, making the nodes clearly visible. ICG dye contrasts between healthy and abnormal tissue, allowing for the removal of the necessary tissue. In the laboratory, the tissues are tested to determine if they are malignant. Original work created by the co-authors in Chem Draw 20.1.

Bargon CA. et al. aimed to compare the sentinel lymph node (SLN) detection rate of ICG fluorescence imaging (Figure 4) with the standard 99mTc-nanocoilloid used in sentinel lymph node biopsy (SLNB) for breast cancer. A total of 102 patients with early-stage, clinically node-negative breast cancer was enrolled and underwent SLNB, first using ICG-fluorescence imaging, followed by confirmation with 99mTc-nanocoilloid using a gamma probe. The primary outcome, the detection rate, was defined as the proportion of patients with at least one SLN detected by either tracer. The results showed that the detection rate for ICG fluorescence was 96.1%, significantly higher than the 86.4% detection rate for 99mTc-nanocoilloid. However, the detection rates for pathological SLNs were identical at 86.7% for both methods, and ICG fluorescence did not increase the detection time, with no adverse events reported. This study concluded that ICG fluorescence demonstrated a higher SLN detection rate than 99mTc-nanocoilloid, with equivalent detection of pathological SLNs, suggesting that ICG fluorescence is a safe and effective alternative for SLNB in early-stage breast cancer patients [107]. Further studies have highlighted the advantages of ICG in breast cancer procedures. For instance, a prospective trial involving 88 patients found that ICG had a detection rate of 96%, compared to 93% for the traditional radioisotope 99mTc, with a combined detection rate of 99%. Importantly, ICG detected all macrometastatic nodes without complications, supporting its role as a viable alternative to radioisotopes for sentinel lymph node biopsy [108].

Another study comparing dual tracer methods found that the ICG–radioisotope combination was safe, with no adverse reactions compared to the blue dye-radioisotope method, which resulted in cases of anaphylaxis and skin tattooing. Despite the higher cost of the ICG method, it was considered a safer and effective alternative for SLNB [109]. ICG’s utility extends beyond SLNB, with studies demonstrating its effectiveness in other breast cancer-related procedures. For example, ICG fluorescence-guided ultrasound was shown to significantly reduce positive frozen resection margins in breast-conserving surgery compared to traditional skin marking, with a lower rate of positive margins and fewer additional resections [110]. Additionally, ICG fluorescence staging has been found to more precisely predict changes in arm volume, fat mass, and lean mass in breast cancer-related lymphedema, providing superior staging compared to clinical exams, though both methods have limitations in predicting these outcomes [111]. Overall, ICG fluorescence continues to show promise in improving the accuracy and safety of various breast cancer treatments, from SLNB to breast reconstruction and lymphedema staging.

### 2.3. Parathyroid Gland Preservation and Postoperative Outcomes Using NIR Autofluorescence and ICG Fluorescence in Total Thyroidectomy

A series of studies explored the use of near-infrared autofluorescence (NIFI) and ICGF imaging to improve the identification and preservation of parathyroid glands (PGs) during total thyroidectomy, with a particular focus on reducing postoperative hypocalcemia and hypoparathyroidism. One randomized prospective study demonstrated that the combination of NIFI and ICGF significantly improved the identification of parathyroid glands (3.83 vs. 3.64, *p* = 0.028) and reduced rates of symptomatic hypocalcemia (6% vs. 17%, *p* = 0.015). Additionally, it was found that identifying at least two well-vascularized parathyroid glands correlated with lower transient hypocalcemia rates and higher postoperative calcium and parathyroid hormone levels [112]. A separate study using ICG angiography also found that this technique improved PG identification and perfusion assessment, reducing the incidence of transient postoperative hypocalcemia from 17.86% in the standard group to 6.67% in the ICG group, suggesting that ICG-assisted thyroidectomy is a safer method for PG preservation [99]. Further analysis of combined autofluorescence and ICGF in 180 patients revealed a lower incidence of transient hypoparathyroidism in the NIFI group (27.8% vs. 43.3%, *p* = 0.029) and a higher rate of PG preservation in situ, supporting the efficacy of these imaging tools in enhancing PG identification and function preservation [113]. Additionally, a noninferiority trial on ICG angiography suggested that postoperative calcium and calcitriol supplementation could be safely omitted in patients with well-perfused parathyroid glands, reducing the need for standard postoperative interventions [114]. Collectively, these studies highlight the potential of NIFI and ICGF as valuable tools for improving parathyroid function preservation and reducing complications following thyroid surgery.

### 2.4. Applications of ICG Fluorescence Imaging in Surgical Precision and Lymph Node Identification

This series of studies explores the application of ICG fluorescence imaging in various surgical procedures, highlighting its effectiveness in improving intraoperative localization and lymph node identification. One study focused on radical esophagectomy for esophageal squamous cell carcinoma, where ICG-near-infrared fluorescence successfully identified sentinel lymph nodes in all patients, with 100% sensitivity, detection rate, and negative predictive value. The ICG-guided approach also led to a significantly higher number of mediastinal lymph nodes being resected compared to traditional methods, suggesting its potential to enhance lymph node detection during esophagectomy [115]. Another study exploring thoracoscopic segmentectomy for lung nodules demonstrated that a real-time, image-guided ICG dual-visualization technique provided a negative resection margin in all patients, ensuring safe resection while preserving pulmonary parenchyma. The technique was found to be free from toxicity or intraoperative complications, supporting its safety and effectiveness [116]. Similarly, a trial investigating ICG fluorescence video-assisted thoracoscopic surgery (FLVATS) for small pulmonary nodule resections found it significantly outperformed white-light VATS (WLVATS), achieving a higher localization rate (87.1% vs. 59.1%) and requiring less time for nodule localization, especially for small ground-glass opacities [117]. Additionally, ICG lymphangiography has proven to be a valuable tool in identifying the thoracic duct during left lateral neck dissection, with improved visualization in 64% of patients compared to 48% with ambient light. This method was particularly successful in patients with prior neck radiation or surgery, offering a safe and effective alternative to traditional techniques, with no postoperative chylous fistulas [118]. These studies collectively highlight the promising role of ICG fluorescence in enhancing surgical precision, improving outcomes, and minimizing complications across a range of procedures.

### 2.5. Evaluating the Impact of ICG Fluorescence Imaging in Colorectal and Cancer Surgery

A series of studies explored the application of ICG fluorescence imaging in colorectal and cancer surgeries, with mixed outcomes in reducing complications and improving surgical precision. A phase 3 trial examining the impact of ICG-guided bowel anastomosis (FGBA) on preventing anastomotic leakage in colorectal surgery found no significant difference in leakage rates between the ICG and conventional surgery groups, suggesting that ICG imaging may not substantially reduce leakage across all colorectal surgeries [119]. In contrast, a trial focused on minimally invasive rectal cancer surgery showed that ICG fluorescence imaging significantly reduced anastomotic leakage rates and reoperation rates, indicating its potential benefit in rectal cancer surgeries [120]. ICG also proved effective in enhancing D3 lymph node dissection in sigmoid and rectal cancer patients, improving the number of lymph nodes harvested, though it did not impact the number of positive nodes [121].

**Figure 5:**
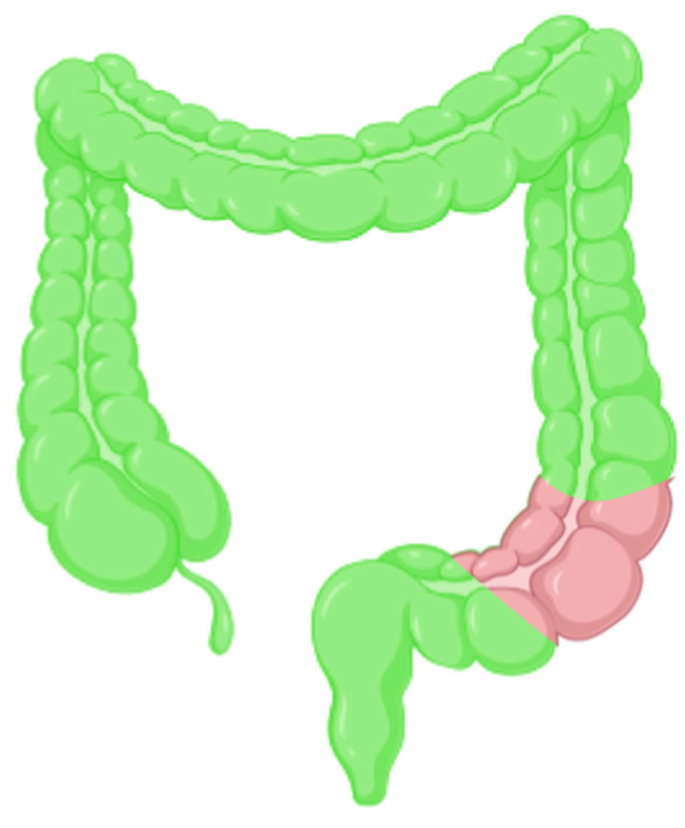
Transrectal ICG angiography imaging the blood supply to the mucosa and anastomosis so that tissue perfusion defects that can lead to anastomotic failure can be detected. Pink color—poor tissue perfusion, green—good tissue perfusion. Original work created by the co-authors in Chem Draw 20.1.

Studies evaluating ICG angiography (Figure 5) for tissue perfusion assessment during colon or rectal resections and high ligation of the inferior mesenteric artery (IMA) showed that while ICG effectively assessed vascularization, it did not significantly reduce anastomotic leaks, though ICG-guided IMA ligation resulted in better lymph node retrieval and no complications [122,123]. Additionally, the MIMIC trial demonstrated that ICG FGS improved the rate of tumor-negative resections in colorectal liver metastasis surgery, providing real-time feedback to enhance surgical precision and outcomes [124]. These findings suggest that while ICG fluorescence imaging shows promise in specific areas, its effectiveness may vary depending on the procedure and patient population.

### 2.6. ICG Fluorescence Imaging in Urological Field and Cancer Treatment

Recent studies have highlighted the effectiveness and safety of ICG fluorescence imaging in various surgical procedures, particularly in oncology. A randomized trial investigating ICG fluorescence-guided inguinal lymph node dissection (ILND) in penile cancer patients showed that the ICG-guided side yielded significantly more inguinal lymph nodes (ILNs) compared to the non-ICG-guided side without increasing complications, making it a promising technique for lymph node retrieval in selected penile cancer patients [125]. Similarly, in robotic partial nephrectomy for renal tumors, ICG fluorescence enabled precise arterial clamping, enhancing tumor resection accuracy and preserving renal function with minimal complications [126]. In kidney transplantation, quantitative ICG fluorescence angiography proved valuable in predicting short-term outcomes like delayed graft function (DGF), with the ICG Ingress parameter effectively correlating with early postoperative kidney function and ischemia times [127]. Another randomized trial compared personalized ICG-guided pelvic lymph node dissection (PLND) with extended PLND during prostate cancer surgery, finding that ICG-PLND resulted in fewer complications and similar oncological outcomes, offering a safer alternative for lymph node staging in high-risk prostate cancer patients [128]. Lastly, a study on low-risk penile cancer surgery showed that NIR fluorescence-assisted inguinal lymph node dissection not only improved lymph node retrieval and reduced surgical complications but also enhanced the detection of metastases, underlining its importance in precise dissection [129]. These studies demonstrate that ICG fluorescence imaging improves surgical precision, reduces complications, and enhances the detection of metastases across various cancer types.

### 2.7. Efficacy of ICG Fluorescence Imaging in Laparoscopic Gastrectomy for Gastric Cancer

A series of studies have explored the efficacy and safety of ICG-guided fluorescence imaging in the context of laparoscopic gastrectomy for gastric cancer (GC), showing promising results. A phase 3, open-label, randomized clinical trial (NCT03050879) demonstrated that ICG fluorescence imaging significantly increased the number of lymph nodes (LNs) retrieved during laparoscopic gastrectomy, with a mean of 50.5 LNs retrieved in the ICG group compared to 42.0 in the non-ICG group (*p* < 0.001). Furthermore, the ICG group exhibited better three-year overall survival (OS) and disease-free survival (DFS) rates (log-rank *p* = 0.015 and *p* = 0.012, respectively) and lower recurrence rates (17.8% vs. 31.0%) [130]. Similarly, a prospective randomized study involving 266 patients found that ICG-guided imaging enhanced the retrieval of perigastric and extra perigastric lymph nodes, with a lower lymph node noncompliance rate (31.8% vs. 57.4%, *p* < 0.001) without increasing complications [131]. Moreover, another study on patients who received neoadjuvant chemotherapy (NAC) showed that ICG-guided lymphadenectomy improved the quality of lymph node dissection, especially in those with measurable baseline lymph nodes [132]. While a phase 2 trial on distal gastrectomy found that NIR ICG visualization helped harvest additional nodes, its clinical benefit in terms of oncological outcomes was modest, with only 39% of patients benefiting clinically [133]. Together, these findings suggest that ICG fluorescence imaging is a valuable tool in improving lymphadenectomy during laparoscopic gastrectomy, contributing to better survival outcomes and enhanced surgical precision.

### 2.8. Use of ICG Fluorescence Imaging in Endometriosis Surgery

This series of studies evaluated the use of ICG fluorescence imaging in the context of endometriosis surgery, particularly for deep infiltrating endometriosis (DIE). One clinical trial focused on the feasibility of ICG to assess the vascularization of the resected area during laparoscopic rectal shaving in 21 patients. The results showed that 81% of patients exhibited very good fluorescence at the rectal shaving site, and no adverse reactions occurred. The use of ICG did not increase the operating time, and in one case, the surgical approach was adjusted based on fluorescence results, with no digestive fistulas reported. This study concluded that ICG fluorescent imaging is feasible and could enhance safety in bowel surgery for deep infiltrating endometriosis [134]. Another study investigated the role of NIR fluorescence with ICG for detecting endometriosis lesions during laparoscopic surgery. While ICG identified 32 additional lesions beyond what white-light imaging detected, its diagnostic value alone was limited, with only one of seven exclusively ICG-identified lesions confirmed as endometriosis. However, the combination of white light and NIR-ICG imaging improved lesion detection, especially for deep infiltrating nodules, aiding their resection (Table 2). The study concluded that while ICG fluorescence is not highly diagnostic on its own, it is valuable in aiding the surgical resection of deep endometriosis lesions, particularly for delineating the margins of these nodules [135].

### 2.9. ICG Visualization and Safety in Laparoscopic Cholecystectomy

Collectively, these studies emphasize the importance of optimizing the ICG dosage for enhanced surgical visualization and improved outcomes in LC. Several other studies have highlighted the diverse applications of ICG in both diagnostic and surgical settings. One study investigated the ICG retention test (ICG-R15) as a predictor for gastroesophageal varices (GOVs) in cirrhosis, finding that ICG-R15 correlated strongly with variceal severity and outperformed traditional diagnostic methods like ARPI and FIB-4 [140]. Another study focused on preoperative laboratory markers that could predict ICG fluorescence intensity during hepatobiliary surgery, developing a laboratory risk score (TLRS) to help adjust ICG dosing for optimal fluorescent imaging [141]. ICG fluorescence navigation was also beneficial in laparoscopic common bile duct exploration (LCBDE) for complex hepatolithiasis, where it led to reduced operation times, lower blood loss, fewer complications, and faster recovery [142]. Furthermore, a novel laparoscopic hepatectomy navigation system (LHNS), combining 3D preoperative models with ICG fluorescence, resulted in less blood loss, lower transfusion rates, and shorter hospital stays, showing promise for real-time surgical guidance [143]. These studies underscore ICG’s versatility and potential to enhance both diagnostic accuracy and surgical outcomes. ICG fluorescence imaging has also proven valuable in improving surgical precision during hepatobiliary surgeries. One study assessed ICG’s role in determining optimal resection margins during hepatectomy for hepatocellular carcinoma, finding that the fluorescence of tumors and the surrounding green zone, indicating clear margins, had high specificity (1.000) and moderate sensitivity (0.706) [144]. Another study explored ICG fluorescence cholangiography in emergency laparoscopic cholecystectomy for acute cholecystitis, aiming to reduce operating time and improve the visualization of Calot’s Triangle, though research in emergency settings remains limited [145]. ICG fluorescence was also integrated into the Critical View of Safety Plus method during laparoscopic cholecystectomy, improving the visualization of critical biliary structures and reducing the need for further dissection [146]. Lastly, a study comparing ICG fluorescence cholangiography (ICGFC) with conventional laparoscopic cholecystectomy found that ICGFC reduced the time to achieve the critical view of safety, particularly in more difficult cases, suggesting its utility in challenging surgeries and for training [147]. These findings highlight the growing role of ICG fluorescence in enhancing safety, precision, and outcomes in hepatobiliary surgeries. In a comparison of two methods for administering ICG during laparoscopic cholecystectomy, intravenous ICG (IV-ICG) and transhepatic ICG injection directly into the gallbladder (IC-ICG), the study revealed that both techniques were effective in identifying key biliary structures. However, IV-ICG demonstrated superior accuracy, especially in identifying the duodenum and common hepatic duct. While both methods showed similar perioperative features and operating times, IV-ICG provided clearer visualization, whereas IC-ICG improved bile duct-to-liver contrast by avoiding hepatic fluorescence. The study concluded that IC-ICG may be more suitable for patients with liver cirrhosis or acute cholecystitis, offering better contrast and fewer hypersensitivity risks [148]. Additionally, ICG fluorescence imaging was compared with conventional fiber-optic imaging in laparoscopic bile duct exploration, where ICG significantly improved bile duct visibility, shortened operative time, reduced intraoperative blood loss, and minimized complications, highlighting its advantages in laparoscopic cholecystectomy [149]. Table 3 presents the applications of ICG in Cervical Cancer and Endometrial Cancer Surgery

### 2.10. ICG in Drug Dosing and Cardiovascular Surgery

ICG has found diverse applications in both pharmacology and cardiovascular surgery, offering insights into liver function and enhancing surgical precision. One study investigated the potential of the ICG clearance test to predict linezolid overexposure in septic patients, comparing its efficacy to traditional liver function markers. By analyzing the relationship between the linezolid trough concentration (Cmin) and ICG-derived parameters, such as the plasma disappearance rate (ICG-PDR) and the retention ratio (ICG-R15), the study found significant correlations. Specifically, ICG-PDR was identified as an independent predictor of linezolid overexposure, with an optimal cutoff value that demonstrated high sensitivity and specificity. These findings suggest that the ICG test, especially ICG-PDR, can be a valuable tool for optimizing drug dosing in septic patients, providing a non-invasive method for assessing hepatic function and improving clinical outcomes [152]. Similarly, ICG videoangiography (ICG-VA) has been effectively utilized in carotid endarterectomy (CEA) procedures to enhance surgical precision. In a study of 44 CEA surgeries, ICG-VA successfully localized plaque sites, assessed blood flow, and identified thrombus formation post-closure in a significant number of cases. The real-time, high-accuracy imaging provided by ICG-VA allowed for more precise evaluation and better decision-making during surgery, thus improving the safety and outcomes of the procedure [153]. Together, these studies highlight the versatility of ICG in both optimizing drug therapies and advancing surgical techniques. Clinical trials and g rants with the Use of ICG are presented in Table 4.

## 3. Discussion

ICG is a water-soluble dye that is now used extensively in several medical specialties, from surgical guidance to diagnostics. The most notable characteristic of ICG, which was approved by the FDA in 1959, is its capacity to fluoresce when exposed to near-infrared light [157]. Because it enables high-resolution imaging of biological structures, this fluorescent feature is essential for assessing tissue viability, organ function, and blood flow. Its clinical uses have expanded dramatically over the years, and new applications are constantly being discovered, especially considering improvements in imaging and surgical methods [158]. Cardiovascular diagnostics is one of the main fields where ICG is frequently utilized. ICG has shown to be especially useful in evaluating cardiac output and blood flow. The dye travels quickly through the body after being injected into the bloodstream and is mostly eliminated by the liver. Clinicians can determine the volume of blood being pumped by the heart and obtain real-time cardiac function information by tracking the blood’s ICG content over time [159]. The non-invasive observation of blood flow through vessels, including coronary arteries, is made possible by this technique, called indocyanine green angiography, and is very helpful for assessing individuals who may have heart disease. ICG is a dependable tool in clinical practice and research because of its capacity to produce comprehensive pictures of blood flow in small capillaries. This is especially true when it comes to tracking the efficacy of cardiovascular medicine treatments or interventions. ICG is useful for imaging the vascular architecture; however, there are some restrictions on how it can be used [160]. For example, the presence of other compounds in the bloodstream, such as certain drugs, might occasionally modify its fluorescence, which can impact imaging accuracy. Furthermore, although ICG is frequently used to measure cardiac output, it might not work as well in some people due to factors like severe obesity or severe liver failure, which can change how quickly ICG is eliminated from the body [161]. These drawbacks emphasize how crucial it is to combine ICG with additional diagnostic methods for more thorough evaluations. Liver function testing is another well-known use for ICG. ICG is removed from the bloodstream by the liver, and the liver’s health can be inferred from the rate at which the dye is removed. ICG clearance may be slower than usual in patients with liver cirrhosis, hepatitis, or other liver dysfunctions, suggesting diminished liver function [162]. To determine whether a patient’s liver is working well enough for surgery, especially in liver transplantation or resection surgeries, the ICG clearance test is frequently performed prior to surgery. It is regarded as a great tool for assessing liver health without the need for more invasive biopsy procedures because it is a non-invasive and rather easy test [163,164]. The ICG clearance test has limitations while being widely used. A few variables, including the patient’s level of hydration, can affect the test’s accuracy by changing the ICG’s volume of distribution and clearance. Furthermore, although ICG is generally regarded as safe, there are some possible dangers, such as allergic responses, especially for patients who have already experienced dye allergies [165]. This indicates that although ICG offers useful information about liver function, it needs to be utilized carefully in some groups. ICG has been an essential technique in ophthalmology for viewing the retina and evaluating diseases such as diabetic retinopathy, AMD, and retinal vascular occlusions [160]. ICG angiography is especially useful for identifying choroidal neovascularization, a defining feature of diseases such as AMD, which is otherwise challenging to identify with conventional imaging techniques [166]. To guide treatment decisions, such as the administration of anti-VEGF medication, which attempts to prevent aberrant blood vessel formation in the retina, it is essential to be able to differentiate between healthy and sick tissue. ICG’s application in ophthalmology is not without restrictions, though [167]. Although fluorescein angiography, another dye used in retinal imaging, provides better images of the superficial retinal layers, it does not reveal as much detail of the deep retinal and choroidal structures. Furthermore, there is a little chance of adverse effects from ICG injections, such as allergic responses or a brief increase in intraocular pressure, which should be carefully evaluated, especially in individuals who already have eye disorders [168,169]. ICG has become a potent intraoperative imaging tool in surgery. During procedures, surgeons can see blood flow, tissue viability, and even lymphatic drainage thanks to its near-infrared fluorescence. This is especially helpful in oncological surgeries, where it can assist in identifying malignant tissues and assessing margins, or in procedures like CABG, where ICG can be used to evaluate the patency of grafts [170]. Additionally, because ICG can be used to map the lymphatic system in real time, a process known as lymphatic mapping, it has demonstrated potential in identifying lymph node metastases in cancer patients. Real-time, high-resolution images are one of the main advantages of ICG-guided surgery since they can enhance surgical accuracy and results [171]. Potential problems, such as locations with inadequate tissue perfusion or weak blood flow, can be detected early by surgeons. ICG incorporation into surgical practice is not without its difficulties, though. One drawback in some situations, especially those with limited resources, is the requirement for specialist equipment to take pictures of near-infrared fluorescence [172]. Furthermore, even though ICG has been demonstrated to be safe for a variety of surgical operations, individuals with liver or kidney impairment should not use it as it may disrupt the dye’s elimination and result in toxicity. With an eye toward the future, ICG’s potential for utilization in more applications keeps growing [173]. The use of ICG in molecular imaging has been investigated recently; it may be coupled to other molecules, like antibodies or nanoparticles, to target particular disease biomarkers. More individualized and accurate imaging methods may result from this strategy, especially in oncology, where early identification of small, confined malignancies could significantly improve prognosis [174]. Combining ICG with other imaging techniques like positron emission tomography (PET) or MRI may improve its diagnostic capabilities and offer a more thorough understanding of biological processes. There are still obstacles to address despite its enormous potential. ICG’s reliance on near-infrared fluorescence imaging, which necessitates specialist equipment that may not be available in all clinical settings, is one of the main obstacles to its broad usage [175]. Although ICG is usually regarded as safe, care should be taken because of the possibility of side effects, especially in patients who already have underlying medical issues. To get over these restrictions and increase the usage of ICG in novel and creative ways, more research and development is required in both imaging technology and dye compositions. Advantages and Limitations are presented in Table 5.

## 4. Summary

In modern medicine, ICG has various applications, particularly in surgical and diagnostic operations. Its versatility is demonstrated in fields such as ophthalmology, cardiology, and oncology, where it aids in tissue perfusion monitoring, surgical guidance, and tumor visualization. ICG provides high-resolution, real-time images during minimally invasive surgery, increasing accuracy and reducing complications. As technology advances, it is expected that the usage of ICG will increase, improving patient outcomes and increasing the safety and effectiveness of many medical therapies. Further research and clinical studies are required to fully explore its potential and overcome any limitations.

## 5. Conclusions

The realm of medical diagnostics and treatments has greatly benefited from ICG’s photoluminescent qualities. More accurate, real-time imaging has been made possible by its capacity to emit near-infrared fluorescence when exposed to light. This has improved the precision of surgical techniques, especially in the areas of tumor detection, blood flow visualization, and lymphatic mapping. By assisting in the identification of crucial structures and reducing the possibility of residual disease, ICG’s use in FGS has improved surgical outcomes. Additionally, its potential for therapeutic uses like photothermal therapy creates new opportunities for focused cancer treatment. Notwithstanding its encouraging potential, there are still issues with optimizing ICG’s long-term effects, pharmacokinetics, and safety. ICG’s uses will probably grow because of ongoing research into its usage in personalized medicine as well as technological developments in imaging and treatment modalities, making it an even more crucial tool in contemporary medical practice. ICG appears to have a bright future in medicine, with more advancements anticipated to increase its influence on patient care, treatment accuracy, and diagnostics.

## Figures and Tables

**Table 1:** The types of nanoparticles that have been explored for encapsulating ICG. The included images were created using PerkinElmer ChemOffice Suite 20.1.

Nanoparticle Group	Short Characteristic	Mechanism	Application
Liposomes 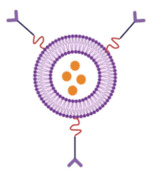	Lipid-based nanoparticles that are perfect for encapsulating ICG because they can include both hydrophilic and hydrophobic compounds. ICG is soluble in water [60].	Liposomes can release ICG in a regulated manner and are biocompatible and simple to make. To target particular cells or tissues, they can also be functionalized with ligands (such as antibodies) [61].	Liposomal ICG formulations are frequently employed in cancer treatment, targeted drug administration, and fluorescence imaging [62].
Polymeric nanoparticles 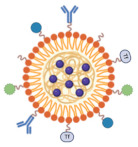	Composed of biocompatible and biodegradable polymers, such as chitosan, polylactic acid, or PLGA. ICG can be encapsulated by these nanoparticles, which also provide controlled drug release capabilities [63].	Because of their stability, ease of functionalization, and continuous release of ICG, these nanoparticles lower the possibility of fast breakdown [64].	They are frequently employed as drug delivery systems to give therapeutic medications and imaging chemicals to tumor locations, as well as for cancer imaging [65].
Dendrimers 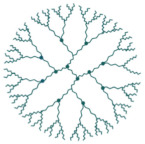	Well-defined, spherical macromolecules that resemble trees and have many branches. To transport ICG, their surface can be readily altered using functional groups [66].	Dendrimers can be made to carry several imaging or therapeutic chemicals at once and have a large surface area for loading ICG. Through surface changes, they may also be able to target particular tissues or cells [67].	Dendrimers are used in combination therapy and targeted cancer imaging to deliver chemotherapeutic drugs and ICG to malignant regions [68].
Inorganic nanoparticles 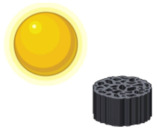	Because of their plasmonic characteristics, gold nanoparticles can intensify fluorescence signals and offer a distinctive contrast in imaging [69]. Silica nanoparticles are easy to functionalize, stable, and biocompatible [70].	These inorganic nanoparticles enable accurate targeting and can increase ICG’s fluorescence [71].	Inorganic nanoparticles are being investigated for phototherapy applications and are employed in integrated imaging modalities like fluorescence and photoacoustic imaging [20].

**Table 2:** Laparoscopic cholecystectomy studies on patients using ICG.

Study	Sample Size	ICG Dosage	Key Findings	Conclusion
Randomized Clinical Trial [136]	195 patients	0.01 mg/BMI, 0.02 mg/BMI, 0.04 mg/BMI	Both 0.02 mg/BMI and 0.04 mg/BMI provided superior biliary visualization, especially of the common hepatic duct. No significant differences between 0.02 mg/BMI and 0.04 mg/BMI.	A dose of 0.02 mg/BMI offers the best balance of efficacy and safety for fluorescent LC.
4K Fluorescent System Study [137]	40 patients	1 µg, 10 µg, 25 µg, 100 µg	Higher doses (25 µg, 100 µg) resulted in stronger fluorescence but increased liver background interference. Doses of 10 µg and 25 µg provided the clearest fluorescence and best bile-to-liver ratio.	A 10–25 µg dose is optimal for real-time fluorescent cholangiography.
Multicenter Trial [138]	55 patients	0.05 mg, 2.5 mg	The low-dose group (0.05 mg) had better bile duct visibility with reduced liver fluorescence. Qualitative assessments were not statistically significant.	Low-dose ICG improves biliary visualization in hepatobiliary surgeries.
Single-Dose Study [139]	64 patients	0.1 mg	A 0.1 mg dose resulted in the highest bile duct-to-liver ratio before cystic duct dissection, providing the best cholangiographic outcomes.	A 0.1 mg dose of ICG is optimal for fluorescent cholangiography during LC.

**Table 3:** ICG in cervical cancer and endometrial cancer surgery.

Study	Objective	Sample Size	Procedure	Findings	Outcome
NIRF Imaging with ICG for Tumor Invasion in Cervical Cancer [150]	Assess the feasibility and accuracy of NIRF imaging using ICG for detecting tumor invasion in cervical cancer	51 patients with FIGO stage IB1-IIA2 cervical cancer	Administered 5 mg/kg ICG 24 h before surgery	NIRF successfully identified tumor invasion in 48 patients. A 95.8% agreement rate for stromal invasion and 100% agreement for surgical margin, parametrial, and uterine corpus involvement.	NIRF imaging with ICG provides accurate, objective, and safe detection of tumor invasion, improving surgical precision in cervical cancer surgery.
Randomized Controlled Trial SLN Mapping using CNPs vs. ICG in Endometrial Cancer [151]	Compare sentinel lymph node (SLN) mapping using carbon nanoparticles (CNPs) and ICG in early-stage endometrial cancer	206 patients	Sentinel lymph node mapping with CNPs and ICG	Both methods had similar detection rates, sensitivity (66.7%), and negative predictive value (NPV). A 100%sensitivity rate and NPV when calculated per hemipelvis or bilateral SLN detection.	CNPs offer a feasible and accurate alternative to ICG for SLN mapping, especially when near-infrared imaging is unavailable.

**Table 4:** Clinical trials and grants.

Study/Trial	Objective	ICG Angiography Role	Findings
Faricimab vs. Aflibercept—Choroidal Neovascularization [154]	Compare efficacy and safety of 6 mg Faricimab vs. aflibercept in CNV patients.	Used to assess vascular changes in the retina and the impact on neovascular processes in CNV.	ICG angiography provided insights into neovascularization and treatment effectiveness.
Aflibercept—Polypoidal Choroidal Vasculopathy [155]	Investigate Aflibercept’s effect on polypoidal choroidal vasculopathy (PCV) associated with CNV.	Employed to visualize and quantify polypoidal lesions, aiding in effective treatment strategy.	ICG angiography helped in visualizing polypoidal lesions and informing treatment decisions.
Polypoidal Subretinal Vascularization [156]	Differentiate and categorize subtypes of polypoidal subretinal vascularization.	Used to differentiate between different subtypes of polypoidal subretinal vascularization for treatment decisions.	Enabled more accurate categorization of vascularization types, improving treatment management.
Sentinel Node Biopsy (Breast Cancer) [155]	Investigate ICG’s role in enhancing sentinel node biopsy accuracy in breast cancer patients.	ICG-based imaging was used to improve sentinel lymph node detection in breast cancer.	ICG imaging improved the accuracy and detection of sentinel lymph nodes.

**Table 5:** Pros and cons of ICG.

**Advantages of ICG in Clinical Applications**	
Real-time visualization	ICG provides real-time imaging capabilities, making it particularly useful in procedures like fluorescence-guided surgery (FGS). It allows surgeons to visualize structures such as blood vessels, tumors, or lymphatic systems during surgery, enhancing precision and reducing the risk of complications.
Non-invasive	The use of ICG for imaging is minimally invasive. When injected intravenously, it allows for the dynamic visualization of tissues without the need for more invasive procedures like biopsies or additional scans, thus reducing patient risk and recovery time.
Versatility	ICG can be applied across various clinical fields, including oncology (tumor detection), cardiology (vascular imaging), hepatology (liver function assessment), and lymphology (lymph node mapping). Its flexibility in application makes it a useful tool across a range of specialties.
High sensitivity and specificity	ICG fluorescence provides high sensitivity for detecting small vessels, tumors, and lymph nodes. This makes it a valuable tool for detecting early-stage diseases, where traditional imaging may not be as effective.
Low toxicity	ICG is generally considered to have low toxicity, as it is quickly eliminated through the liver and excreted via bile. This makes it a safer contrast agent compared to other dyes, which can have more harmful side effects.
Guiding minimally invasive procedures	In endoscopic or laparoscopic surgeries, ICG fluorescence helps surgeons better navigate tissues and organs, improving surgical precision in minimally invasive procedures.
**Limitations of ICG in clinical applications**	
Limited penetration depth	One of the major limitations of ICG is its relatively shallow tissue penetration. ICG fluorescence is often limited to superficial structures, which may reduce its effectiveness in visualizing deeper tissues or organs, especially in larger patients or obese individuals.
Dye interference	The fluorescence signal from ICG can sometimes be obscured by tissue autofluorescence or interference from other dyes. This can complicate the interpretation of images, especially in tissues with high autofluorescence, such as adipose tissue or certain tumor types.
Dependency on technology	The success of ICG in clinical practice is highly dependent on the specific fluorescence imaging equipment available. Not all medical centers may have access to the advanced, high-resolution imaging systems required to capture and interpret ICG fluorescence accurately.
Short half-life	ICG has a relatively short half-life in circulation (approximately 3–4 min), which means it is only effective for real-time imaging and limits its use for more extended monitoring or the continuous tracking of anatomical structures over long periods.
Liver dysfunction impact	Since ICG is primarily cleared by the liver, patients with liver dysfunction may not be able to properly metabolize and clear the dye, which could lead to false readings or an inability to obtain clear imaging results in such patients.
Lack of specificity in some applications	ICG alone does not have inherent specificity for certain tissues or conditions. For example, while it can identify lymphatic vessels, it does not differentiate between malignant and benign tissue unless combined with other agents or imaging modalities.
Small sample sizes	Many of the studies investigating ICG have small sample sizes, which may limit the generalizability of results. The limited data make it challenging to draw definitive conclusions about its clinical efficacy across diverse patient populations.
Lack of comparative studies	While ICG has shown promising results, there are few comparative studies that directly assess the efficacy of ICG against other imaging techniques like MRI, CT scans, or other contrast agents. This limits the ability to determine the optimal clinical context for ICG use.
Long-term safety and efficacy	More long-term studies should be conducted to assess the safety and effectiveness of ICG, particularly in the context of chronic or recurrent diseases. This includes exploring any potential side effects or complications from repeated ICG use.

## Data Availability

All data have been included.

## Abbreviations

The following abbreviations are used in this manuscript:

AMD	Age-related macular degeneration
CNPs	Carbon nanoparticles
CEA	Carotid endarterectomy
CNV	Choroidal neovascularization
DIE	Deep infiltrating endometriosis
DGF	Delayed graft function
EC	Endometrial cancer
FGS	Fluorescence-guided surgery
FLVATS	Fluorescence video-assisted thoracoscopic surgery
ICGFC	ICG fluorescence cholangiography
FGBA	ICG-guided bowel anastomosis
GC	Gastric cancer
ICG-VA	ICG video angiography
ICG	Indocyanine green
ICGA	Indocyanine green angiography
IMA	Inferior mesenteric artery
IV-ICG	Intravenous ICG
LC	Laparoscopic cholecystectomy
LCBDE	Laparoscopic common bile duct exploration
LHNS	Laparoscopic hepatectomy navigation system
LNs	Lymph nodes
NPV	Negative predictive value
NIR	Near-infrared
NIFC	Near-infrared fluorescence cholangiography
NIFI	Near infrared autofluorescence
NAC	Neoadjuvant chemotherapy
OS	Overall survival
PG	Parathyroid gland
PLND	Pelvic lymph node dissection
PTT	Photothermal treatment
ROS	Reactive oxygen species
SLN	Sentinel lymph node
SLNB	Sentinel lymph node biopsy
WLVATS	White-light video-assisted thoracoscopic surgery
